# Successfully addressing non-compliance with behavioral and social infection control measures is a critical component in management of healthcare worker COVID-19 outbreaks: learning outcomes from the first staff outbreak in the main maternity hospital in Qatar

**DOI:** 10.3389/fpubh.2025.1534421

**Published:** 2025-09-01

**Authors:** Hawabibee Mahir Petkar, Bonnie George, Eman Mostafa, Imelda Caseres-Chiuco, Mahmoud Mohamed, Andrew Jeremijenko, Peter Valentine Coyle, Hebah Atef Mohammad AlKhatib, Fatiha Benslimane, Meryem Bensaad, Bayan Al-Barghouthi, Zaid Mahir Petkar, Jameela Al-Ajmi, Hilal Al Rifai, Huda Saleh

**Affiliations:** ^1^Microbiology Division, Department of Laboratory Medicine and Pathology, Hamad Medical Corporation, Doha, Qatar; ^2^Department of Quality and Patient Safety, The Women’s Wellness and Research Center, Hamad Medical Corporation, Doha, Qatar; ^3^Research Center, The Women Wellness and Research Center, Hamad Medical Corporation, Doha, Qatar; ^4^Staff Medical Centre, Hamad Medical Corporation, Doha, Qatar; ^5^Virology Division, Department of Laboratory Medicine and Pathology, Hamad Medical Corporation, Doha, Qatar; ^6^Biomedical Research Center, Qatar University, Doha, Qatar; ^7^Genomics Core, Weill Cornell Medicine – Qatar, Doha, Qatar; ^8^Computer Science Undergraduate, Kings College, London, United Kingdom; ^9^Corporate Infection Control, Hamad Medical Corporation, Doha, Qatar; ^10^The Women’s Wellness and Research Center, Hamad Medical Corporation, Doha, Qatar

**Keywords:** COVID-19 outbreak, healthcare workers, SARS-CoV-2, social and behavioral factors, outbreak management

## Abstract

**Background:**

Nosocomial healthcare worker (HCW) SARS-CoV-2 outbreaks are well recognized. Contact tracing, use of surgical masks, hand hygiene and social distancing can prevent spread. Social and behavioral factors play an important role in outbreak control. We provide an integrated report on management of our first outbreak and lessons learned.

**Methods:**

Demographic and test result information was extracted from the outbreak report. Infection control practices were audited using a standardized behavior assessment tool. Exposure risk was ascertained using World Health Organization definitions. Cases were identified by reverse transcriptase polymerase chain reaction (RT-PCR) or by seroconversion. Whole genome sequencing was performed on RT-PCR positive cases. Statistical analyses were performed in RStudio. Incidence rates and relative risk were used as measures of effect.

**Results:**

Almost 10% of HCWs developed infection; high risk exposures had a statistically higher risk. All isolates were clade 20C. Consistent with the hypothesis, the epidemiological curve showed a mixed outbreak, initially common source, with subsequent sporadic cases possibly from environmental contamination. Interventions: focused on contact tracing and strict compliance with social distancing, PPE use, hand hygiene and environmental cleaning, supported by rigorous audits. Lessons learned: root cause was a symptomatic HCW reporting to work in breach of policy. Contributing factors: failure to challenge the breach, lax managerial oversight, lounge overcrowding and insufficient cleaning staff.

**Discussion:**

Management required a multi-pronged approach. Full delineation of the outbreak required contact tracing, and correlation of epidemiological information with Ct values, whole genome sequencing and serology. Strategies to address social and behavioral factors should be devised considering the local institutional culture Good leadership, ‘speaking up’ for patient safety and linking individual IPC practices to annual evaluations are effective measures.

## Introduction

COVID-19 disease, caused by the SARS-CoV-2 virus, has caused significant mortality and morbidity worldwide (1). The healthcare industry has one of the highest incidences of COVID-19 (2). Frontline HCWs have a 12-fold increased risk compared to community individuals, with inpatient HCWs facing an even greater 24-fold increased risk (3).

Hospital outbreaks affecting patients and staff are well documented (4); a systematic review (4) showed that in 40% of outbreaks, the index case was a HCW, with a patient being the source in only 22.9% of outbreaks. HCWs accounted for 45% of total infected individuals, patients accounted for 51.6% and the remaining comprised either caregivers or visitors (4). Nosocomial outbreaks confined to HCWs may represent an underestimated transmission risk (5). Many are caused by failure to comply with institutional infection control procedures to prevent spread (5). A study of four outbreaks in a university hospital (5) showed that multiple unprotected contacts between infected HCWs caused the outbreak; a strict implementation of physical distancing and mandatory masking was able to terminate all of them. In another study, 79% of hospital-acquired HCW infections were determined to be staff-to-staff transmission, often from unrecognized infection (6). Another study (7) found that working with isolated COVID-19 patients did not increase the risk of COVID-19 but working shifts with presymptomatic healthcare coworkers did.

Strict infection prevention and control (IPC) precautions can help prevent spread in a healthcare setting. Contact tracing, use of surgical masks, hand hygiene and social distancing have been demonstrated to be effective in preventing spread amongst HCWs (8, 9). Early identification of symptomatic workers is an essential factor to avoiding nosocomial clusters (10). Ibiebele et al. (11) found that transmission of COVID-19 to HCW is low with close adherence to PPE guidelines; lapses in infection prevention practices, including dining together, especially at times when COVID-19 is circulating widely in the community increase the risk of exposure and subsequent transmission to HCWs. There are a number of factors (12) adversely affecting HCW compliance with social and behavioral infection control measures during emerging infectious disease outbreaks. These include working outside of emergency or intensive care settings, not working with confirmed infection cases, inconsistent or unclear infection control guidance, lack of concern about risk of infection, lack of monitoring by superiors and observed non-compliance of colleagues. Training and education may not significantly improve compliance in this context possibly due to the content of the training, e.g., focus on ‘how’ rather than ‘why’ or the fact that many of these identified factors are not amenable to training and need a ‘leading by example’ approach from staff in authority (12).

Therefore, lessons need to be learned from the management of outbreaks, including understanding the root causes and contributing factors including social and behavioral. This is key to developing effective preventive strategies to improve compliance going forward. We describe the epidemiology and management of our first nosocomial HCW outbreak. We review the utility of environmental screening, viral sequencing, and serological follow up testing in this outbreak. We also highlight the lessons learned and strategies developed to tackle poor compliance, including those arising from social and behavioral factors. The measures taken in our setting were highly successful at preventing further nosocomial outbreaks and our experience would be of benefit in other similar outbreak situations.

## Setting

The Women’s Wellness and Research Center is the largest women’s hospital in the State of Qatar. The operating suite consists of two second-floor theatres for emergency and five third floor theatres for elective surgeries. There are female and male changing rooms on both floors, a separate nurse and doctors’ lounge on the third floor and a single lounge on the second floor.

At the time of the outbreak, all staff had already completed their annual organizational statutory and mandatory IPC training e-modules and had additionally received face-to-face training on the organization’s infection control measures against COVID-19. These included the appropriate use of personal protective equipment (PPE), rigorous hand hygiene, adequate social distancing in all clinical and non-clinical areas and the necessity for HCWs developing respiratory symptoms and/or fever to report to the staff occupational health clinic (OHC) for testing and clearance before reporting for work. According to policy at the time, return to work was allowed only after a negative result and OHC clearance. Positive staff were mandated to be on 10 days of sick leave before returning to work.

## Methods

### Study design

Retrospective observation study of the epidemiology of an outbreak, with report of management and lessons learned.

### Population

HCWs working in operating suites on both floors.

### Ethical approval

This was received from the Medical Research Council of the Hamad Medical Corporation, State of Qatar (MRC MRC-01-21-121). The study was reviewed and approved by the organization’s institutional review board.

### Definitions

(a) Case: Any HCW working in the operating suites who had been in contact with or had shared the same suite facilities with the presumed index case or subsequently identified positive cases and tested positive for SARS-CoV-2 by reverse-transcriptase polymerase chain reaction (RT-PCR) or demonstrated seroconversion within 30 days of exposure, without any other known exposure outside the outbreak setting. (b) Probable case: Any HCW working in the operating suites who had been in contact with or had shared the same suite facilities with the presumed index case or subsequently identified positive cases and had symptoms consistent with COVID-19 disease but had a negative RT-PCR and no evidence of seroconversion within 30 days of exposure.
Contact: Any HCW with an exposure to the presumed index case or any subsequently identified cases.
High and low risk exposures: This was based on the World Health Organization (WHO) interim guidance at the time of the outbreak (13). Briefly, face-to-face contact with the case at less than two meters for more than 15 min without recommended personal protective equipment (PPE) i.e. eating together, talking or otherwise interacting without masks was considered high risk exposure. Face-to-face contact with a case at more than two meters for less than 15 min or close interaction but wearing recommended PPE, i.e., staff who had used the lounge area at different times to the index case or had no close contact within the lounge area or had close contact but had masks on were considered low risk.
Recommended PPE: Per organization policy at the time of the outbreak-scrubs, surgical gown and mask within the operating theatre and surgical masks and scrubs elsewhere in the suite.

### Data collection

Demographic and test result information was extracted anonymously from the outbreak investigation report. Infection control practice compliance was assessed through observation audits and staff interviews using the organizational Behavioral Compliance Assessment Tool. Briefly, this tool evaluates adherence to 5 key infection prevention and control measures.

1. *Hand Hygiene Compliance*—Assessing frequency and technique of handwashing, including the use of alcohol-based rubs.
2. *Recommended PPE Compliance*—Monitoring mask usage within unit premises during staff interactions.
3. *Social Distancing Compliance*—Observing adherence to appropriate physical distancing among colleagues and patients, especially in non-clinical areas.
4. *Surface Disinfection Practices*—Evaluating the regularity and effectiveness of surface cleaning using appropriate disinfectants.
5. *Behavioral Motivation Factors*—Assessing staff perceptions of encouragement and support in following IPC protocols.

Information on root cause analysis and identification of the root cause/s of the outbreak, contributing factors and lessons learned was extracted from the final integrated outbreak-lessons learned report.

### Statistical methods

Binary outcomes were analyzed with frequency and percentage. Incidence rates were calculated overall and by HCW category. Relative risk (RR) was calculated with 95% confidence intervals. Normally distributed continuous variables, i.e., Ct values were analyzed as mean and standard deviations and compared by ANOVA test across three different time periods. All analyses were performed in RStudio.

### Laboratory methods

All tests were undertaken in accordance with manufacturer’s instructions.

1. RT-PCR: Combined nasopharyngeal and throat swabs collected in universal transport medium were tested on a Roche Cobas 6800 system using the Cobas SARS-CoV-2 Test assay (Roche, Switzerland) that targets the ORF1ab and E-gene regions.
2. Antibody tests were performed using the Roche Elecsys Anti-SARS-CoV-2 (Roche, Switzerland) electrochemiluminescence immunoassay that uses a recombinant protein representing the nucleocapsid (N) antigen of SARS-CoV-2. Qualitative results were generated, i.e., reactive: cutoff index ≥ 1.0 vs. non-reactive: cutoff index < 1.0.
3. Whole genome sequencing (WGS): RNA was extracted using NucleoSpin RNA Virus isolation kits (Macherey-Nagel). The ARTIC Network SARS-CoV-2 sequencing protocol and V3 primer amplicon set were used for sequencing SARS-CoV-2 near full genome on Oxford Nanopore’s GridION platform.
4. Environmental testing: High touch surfaces, e.g., switches, doorknobs, fridges, medication trolleys, keyboards, couches and toilet seats as well as air vents and brooms were swabbed using sterile HydraFlock™ swabs with polystyrene handle (Puritan Medical Products, Guilford, Maine, United States of America) in the following areas:
  2^nd^ and 3rd floor anesthesia preparation rooms.
  2nd floor lounge and 3rd floor nurse’s and doctor’s lounges.
  2^nd^ and 3rd floor male and female changing rooms.
  Swabs were placed in molecular preservative DNA/RNA shield (Zymo Research, Irvine CA, USA Cat. No. R1100-250) and transported in thermally insulated coolers to the laboratory.
  Extraction was performed using Quick-RNA Viral Kits (Zymo Research, Irvine CA, USA Cat. No. R1041). The detection of SARS-CoV-2 in the extract was performed using SARS-CoV-2 (2019-nCoV) CDC qPCR Probe Assay Research Use Only (RUO) kit (Integrated DNA Technologies, IDT, Coralville, IA, USA Cat number 10006713). All RT-qPCR amplifications were performed in 20 μL reaction mixtures using Luna Universal Probe One-Step RT-qPCR Kit (New England BioLabs, Ipswich, MA, USA Cat number E3006E). RT-qPCR assays were performed in 96-well plate on an Applied Biosystems 7500 Fast Real-Time PCR Instrument (Applied Biosystems, CA, USA).

The results were expressed in cycle threshold (Ct) values as:

Below 30: strong positive for virus presence (potentially infectious).
30–35: weak positive for virus presence but at low levels and likely not infectious.
36–40: borderline positive for virus presence potentially indicative of recent past presence of virus particles (someone infectious in that area days ago or for a short time) and clearing out the fragments of the virus not complete.
Above 40: negative.

## Results

### Cases

RT-PCR and antibody results are shown in Table 1. A total of 262 HCWs were tested. Twenty-six cases were identified with overall incidence rate of 9.9%. The highest incidence rates were found amongst anesthesia technicians, which is the same HCW category as the index case. Fourteen cases were positive by RT-PCR and twelve were detected by seroconversion. The presumed index case was the first to be identified; he also was the first to develop symptoms, at least 4 days prior to the subsequent symptomatic cases. There were no probable cases as symptomatic HCWs with initial negative RT-PCR were retested after 24–48 h and all were positive on repeat testing.

**Table 1:** SARS-CoV-2 RT-PCR and antibody results by HCW category.

HCW category	Number exposed	Total cases	Incidence rate	RT-PCR positive/ no tested	Antibody reactive /no tested*
Anaesthetists	35	3	8.6%	2/35	1/23
Anaesthesia technicians	26	9	34.6%	7/26	2/19
Nurses	74	6	8.1%	4/74	2/69
Obstetricians	77	3	3.9%	1/77	2/50
Medical students	14	0	0%	0/14	Not tested
Nursing aides	16	2	12.5%	0/16	2/16
Housekeeping staff	20	3	15%	0/20	3/20
Total	262	26	9.9%	14/262	12/197

*Excludes 14 RT-PCR positive cases.

**Figure 1:**
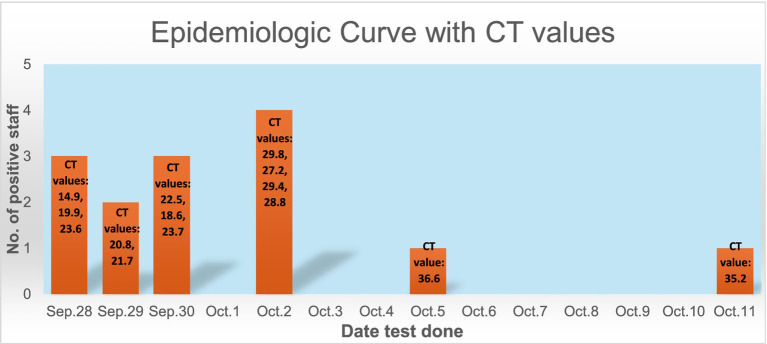
Epidemiologic curve with CT values.

**Figure 2:**
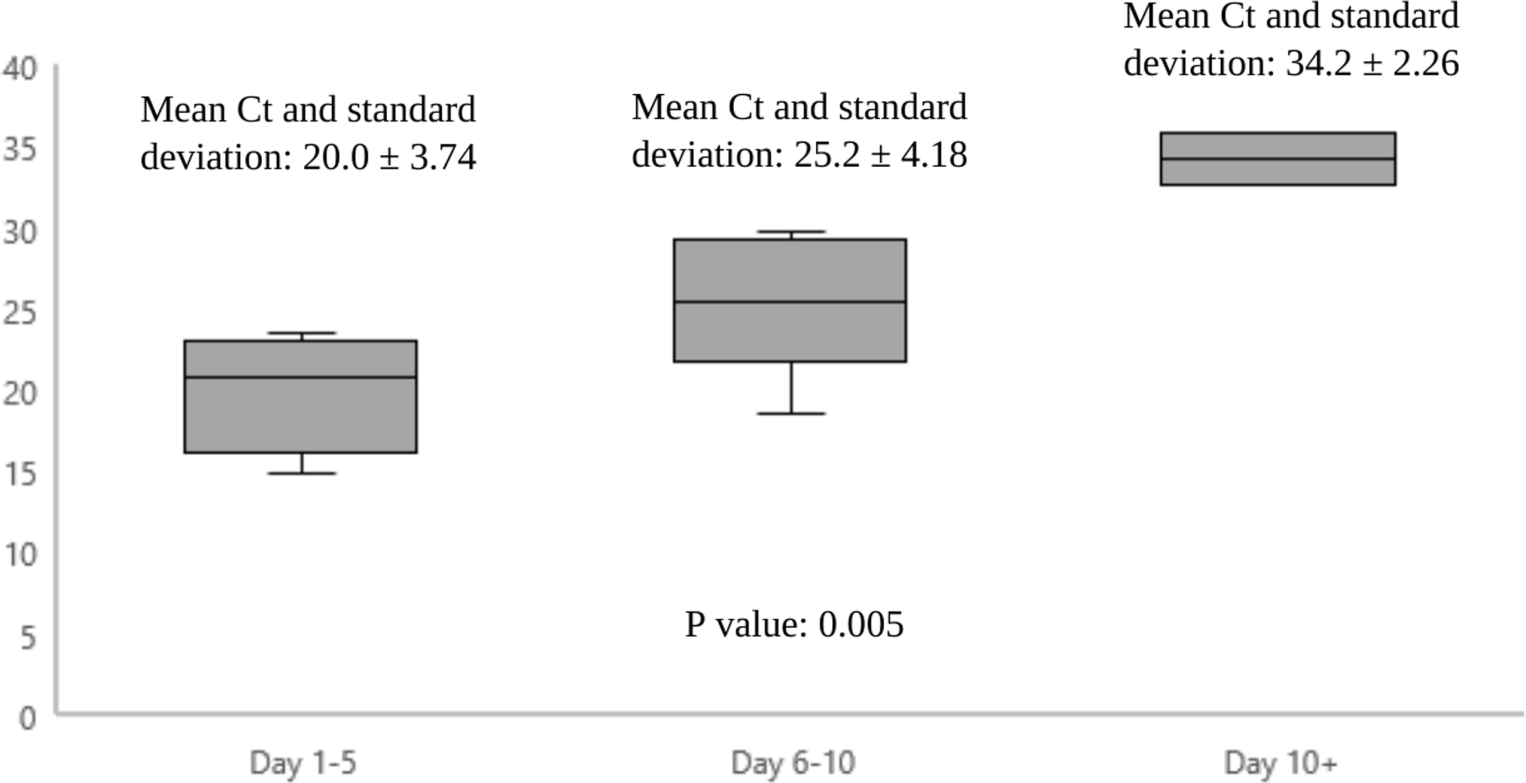
Mean Ct values over the course of the outbreak.

Figure 1 shows the epidemiological curve and Ct values of RT-PCR positive cases. The appearance is of a mixed outbreak with: (1) an initial clustering of cases suggestive of a common source, (2) another peak a couple of days later probably due to identification of propagated cases, and (3) a couple of sporadic cases identified over the next 10 days suggesting intermittent exposure due to possible environmental contamination or identification of late infections. The Ct value of the presumed index case was 19.9. Ct values of cases significantly increased during the course of the outbreak (Figure 2), with the lowest values being found in the initial cluster followed by the propagated cases. As Ct values are inversely related to viral loads, this would imply that cases identified earlier had higher viral loads and were therefore more infectious (14).

Table 2 shows cases by exposure risk. High risk exposures carried a statistically significant risk of developing infection (RR of 2.38). Anesthesia technicians and anesthetists had the highest number of high-risk exposures; unsurprisingly, these were the staff categories that the index case had most contact with on a daily basis. The RR for both categories was <1 but the 95% confidence interval ranges were wide, with the lower limit of <1 and the upper limit >1 so no conclusions on risk could be drawn. The relative risk was highest in nurses, followed by obstetricians.

**Table 2:** Cases by exposure risk.

HCW category	High risk exposures	Low risk exposures	Cases		Relative risk* (95% confidence interval)
			High risk	Low risk	
Anesthetists	28	7	2	1	0.5 (0.05–4.76)
Anesthesia technicians	25	1	8	1	0.64 (0.14–2.86)
Nurses	11	63	2	3	3.82 (0.72–20.39)
Obstetricians	18	59	1	2	1.64 (0.16–17.04)
Medical students	0	14	0	0	N/A
Nursing aides	0	16	0	2	N/A
Housekeeping staff	0	20	0	3	N/A
Total	82	180	13	12	2.38 (1.13–4.98)
% positive			15.8%	7.2%	

N/A, not applicable as at least one field is 0. *Relative risk >1.00 considered to be statistically significant.

### Sequencing results

All sequences belonged to lineage B.1 (Clade 20C). The circulating clades within the country at the time of the outbreak were Clade 20C (>95%) and clades 20A and 20B.

### Environmental screening results

All the areas swabbed were found to have a Ct value >40 except for the doctor’s lounge vent (third floor), third floor anesthesia room vent and the second-floor lounge broom which all had a Ct value of >35 but <40.

### Behavioral compliance assessment audit results

Compliance rates before and at the end of the outbreak are shown in Table 3. The areas where significant non-compliance was seen were in adherence to recommended PPE (mainly use of surgical mask in non-clinical areas), social distancing and disinfection practices; all areas that are known to increase the risk of spread. All these areas showed steadily improving compliance with levels reaching >90% by the end of the outbreak.

**Table 3:** Behavioral compliance assessment results.

IPC measure	At the start of the outbreak	At the end of the outbreak
Hand Hygiene Compliance	88%	93%
Recommended PPE Compliance	65%	95%
Social Distancing Compliance	70%	100%
Surface Disinfection Practices	80%	100%
Behavioral Motivation Factors	90%	100%

#### Hypothesis generation

The presumed index case (or an undetected close contact) was the initial source, directly infecting close contacts who then propagated the infection to other close contacts in the suite. Where no high-risk exposure occurred, it was postulated that recent environmental contamination, when the virus would still have been viable, had played a role in acquisition.

### Observations and interventions

The first case, an anesthesia technician, developed flu-like symptoms at home on 23rd September 2020. He reported for his shift on the second-floor theatres on 25th September as he was unable to get an appointment with the OHC and felt better. On 28th September, his symptoms worsened, and he was finally instructed to go to the OHC where he tested positive.

**Figure 3:**
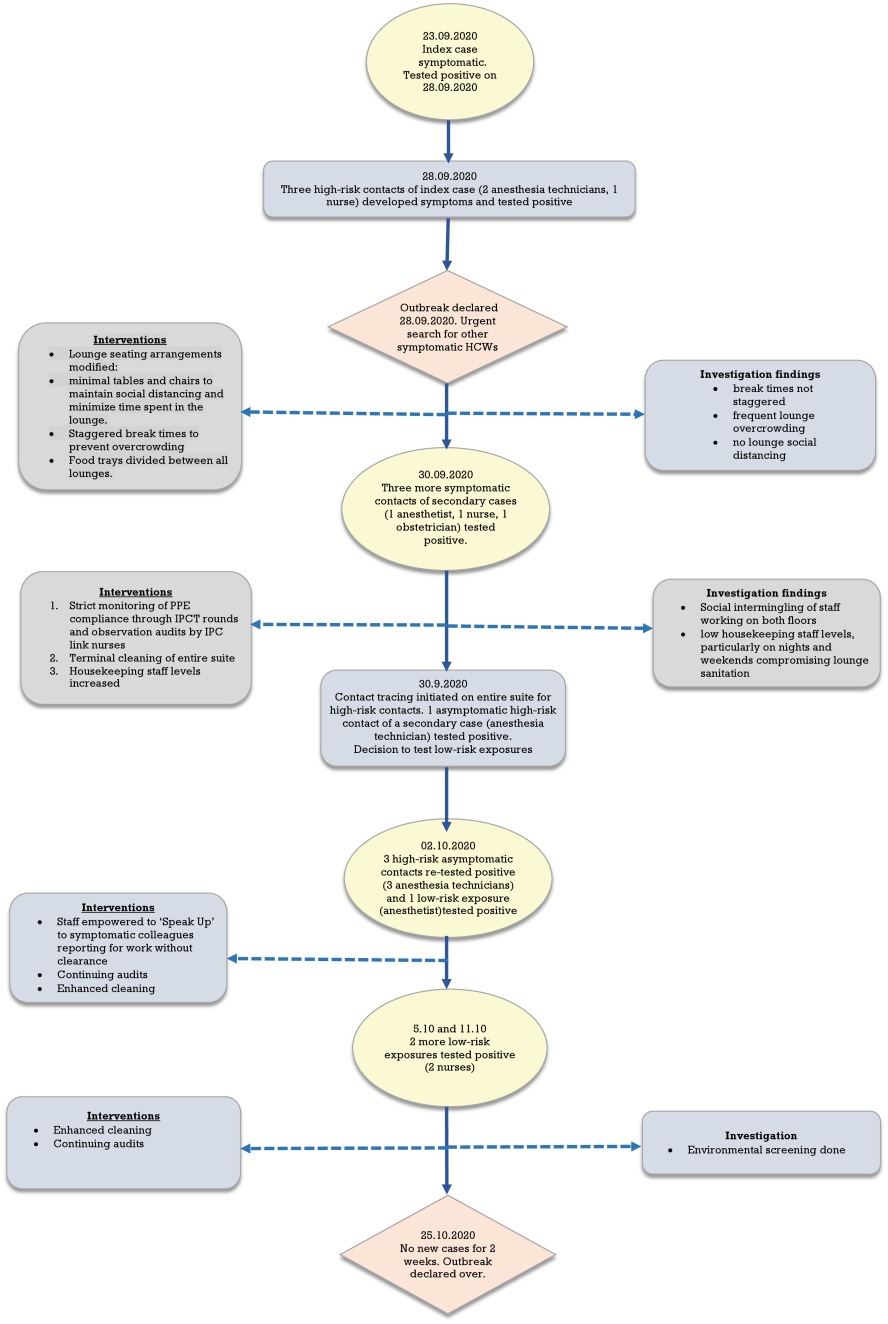
Flowchart showing timescale and interventions.

Figure 3 shows a flow chart summarizing exposures, testing, and control interventions over the course of the outbreak. Initially, testing was undertaken for contacts of the index case and then subsequently identified secondary cases. Subsequently, due to the high number of cases on both floors and the lounges identified as the probable exposure locations, it was decided to test all staff with low-risk exposures as well. Contacts were taken off work pending negative RT-PCR results. Those testing negative on the first test were retested 48–72 h later and could return to work if the second test was negative. HCWs with low-risk exposures were allowed to continue working with the recommended PPE whilst awaiting RT-PCR results with advice to report immediately to the OHC should symptoms occur. They were tested only once unless they developed symptoms. HCWs with negative RT-PCR results were tested a month after exposure for any seroconversion to determine the full extent of the outbreak. Environmental screening to check for residual contamination was done after the occurrence of two sporadic cases in low-risk exposures despite the interventions.

Interventions were made depending on the findings of the investigation as they became available. Briefly, initial interventions were focused on measures to maintain social distancing and reduce overcrowding in lounges alongside improving compliance with PPE and enhanced cleaning focusing on common areas such as the lounge, changing rooms and toilets. This was followed by the empowering of staff to challenge non-compliant behavior of colleagues. Throughout, compliance with interventions was monitored through regular IPCT rounds and audits by link-nurses.

All patient contacts were risk assessed for any potential exposure; it was determined that HCWs and patients had complied strictly with the recommended PPE policies during interactions in the suite, so the risk was considered negligible. However, a record of patients cared for by the positive staff was kept as a precautionary measure as the investigation was still on-going.

The outbreak was declared over on 25th October after 2 weeks with no new cases.

### Lessons learned

The root cause of this outbreak was deviation from organizational policy, i.e., a HCW with symptoms compatible with possible COVID-19 disease reported to work and so failed to follow preventive measures to limit spread. This happened despite bespoke training and an intense awareness campaign for all staff over the preceding months. A number of factors contributed to spread: failure to challenge the HCW sooner to report to the OHC, disregard for organization policy in the staff lounge areas, uncontrolled use of lounge areas, lax oversight of compliance at a unit level and insufficient numbers of housekeeping staff. The latter may have resulted in persisting short-term environmental contamination from infected staff, contributing to spread in HCWs with low risk exposures.

Of concern was how to address the social and behavioral aspects as a matter of priority to prevent future outbreaks. This was addressed in a number of ways that tightened and expanded on existing organization IPC policies and procedures as follows: (1) Findings and lessons from the outbreak were extensively communicated to all hospital staff through daily safety huddles, morning handover sessions, leadership huddles and via email alerts. (2) Senior leaders were encouraged to take a more visible role in promoting safe practices. (3) Infection control safe practices were integrated into HCW annual competencies. (4) Audit data on individual compliance was incorporated into annual individual Ongoing Professional Practice Evaluation (OPPE) evaluations. (5) Staff were empowered to ‘Speak Up’ and challenge colleagues who were in breach of policies, without fear of retribution. All these measures were strengthened by objective compliance data obtained through regular audits, both overt and covert through secret auditors. These showed consistently high compliance throughout the rest of the pandemic.

## Discussion

### Hypothesis evaluation

We consider the hypothesis to be confirmed. The cases identified in the initial 2–3 days had direct exposure to the presumed index case but all developed symptoms a few days after the index case. Cases identified in the second peak had direct exposure to these HCWs but not to the index case, suggesting propagated cases. Ct values are inversely related to viral loads (15); therefore, the significantly lower Ct values of the index and secondary cases would have made them highly infectious. The last 2 cases had no direct contact but had used the lounges. Environmental contamination would have been high, exacerbated by the reduced frequency of cleaning which was likely to have been the source. Saulnier et al. (16) assert that shared meals and drinks represent an additional transmission route at gatherings; significant quantities of virus dispersed by large droplets during discussions could explain group contamination either directly or indirectly after deposition on surfaces, food, drinks, cutlery, and several other soiled vectors. Poor tearoom protocols are known to contribute to HCW outbreaks. In an outbreak on a COVID-19 ward, the incidence of HCW infection significantly reduced after changes were made to the protocols around use of staff tearooms, without any change in PPE, ventilation or patient occupancy of the ward (6). Another alternative explanation of these 2 cases could have been late identification. Their Ct values were much higher, more consistent with later stages of infection when infectiousness is low (14).

### Cases

The highest number of high-risk exposures were found in the cohort most closely working with the index case, i.e., anesthesia technicians and anesthetists. Though the incidence rates reflected this close association, RRs were <1.0 for both categories. We suggest that given the 95% confidence intervals for both these categories were quite wide, ranging from as low as 0.05 to as high as 4.76, an increased risk cannot be excluded.

### Usefulness of laboratory tests

WGS is a useful tool for confirming and investigating outbreaks in HCWs to determine transmission and spread particularly when community transmission is high and there are many circulating clades (17). In our case, the outbreak clade accounted for >95% of circulating community strains and therefore could have represented a coincidental finding., For confirmation, it is important to correlate WGS data with epidemiological information on potential chains of transmission, i.e., identifying contacts and linking this to cases. Ct values can be useful in this situation and may inform when new cases stop occurring. Low Ct values are more likely to reflect recent acquisition; if cases are identified later on in an outbreak and have higher Ct values, it suggests acquisition likely took place earlier in the outbreak rather than ongoing transmission. Salvatore et al. (18) have shown that Ct values are significantly correlated with symptom onset. Within 7 days after symptom onset, the median Ct values are 26.5, compared with a median Ct value of 35.0 occurring 21 days after onset. Demonstration of seroconversion is helpful in ascertaining the full extent of an outbreak, particularly as follow up RT-PCR testing is not always feasible for all exposures.

Environmental testing was undertaken considerably late in our outbreak following on-going sporadic cases. By this time, terminal cleaning had already taken place and enhanced cleaning was on-going. Whilst interpretation of Ct values near the detection threshold warrant cautious interpretation, the finding of possible residual virus in the air vents and the broom suggests care needs to be taken to thoroughly disinfect hard to reach areas such as air vents and the need to dispose of items used for cleaning and disinfecting. SARS-CoV-2 particles can retain infectivity in aerosols for up to 16 h at room temperature (19). The virus can survive on various environmental surfaces and be a risk for inoculation of the mucous membranes of the nose, eyes, or mouth (20, 21). Disinfection of surfaces with 0.1% or 0.5% hypochlorite solution effectively eliminates the virus (22). SARS-CoV-2 is spread by the droplet and air-borne routes, with the latter being particularly important in poorly ventilated or crowded indoor settings (20). However, there is currently no evidence of human infection caused by infectious aerosols distributed through ventilation ducts.

### Audit results

Audits are a well-recognized tool to improve compliance with IPC measures and improve patient safety in hospital settings (23). By using a standardized tool that objectively measures compliance and feedback the findings at a unit and individual level, we were able to make consistent improvements. Only one much smaller nosocomial HCW outbreak involving 20 staff was seen in the hospital 4 months later, with no further outbreaks to date.

### Lessons learned

Our biggest challenge was addressing the social and behavioral factors leading to at-risk behavior and non-compliance outside the patient setting. We decided to address this in ways that are evidence-based yet practical and sustainable in the long term to produce consistent improvements. HCWs ability to ‘Speak up’ freely to raise concerns around patient safety and quality of care has been demonstrated to have a preventive effect on human errors or to improve technical and system deficiencies (24). A ‘Speak Up’ campaign had been introduced in our hospital but had been disrupted by the pandemic. This was re-introduced, expanded to include challenging at-risk behavior by HCWs in all hospital areas and incorporated into routine work practices. Research has shown that factors promoting or hindering ‘speaking up’ broadly fall under categories of (a) hierarchical, interdisciplinary and cultural relationships and (b) HCW perception of psychological safety to express opinions freely (24). Addressing many of these factors requires taking into account the local, cultural milieu as well as research-derived evidence when developing strategies and interventions (25). Our campaign set clear expectations of responsibility on all HCWs to speak up when they saw colleagues in breach of IPC precautions without fear of intimidation and demonstrated on-going commitment from senior management towards the success of the campaign. Additionally, we linked outcomes of audits of individual IPC practices to each HCW’s annual appraisal and performance evaluation of professional practice, thus continually highlighting the importance of having consistently good IPC practices. This was supported by a commitment from senior leadership, multi-disciplinary teamwork and clear communication of expectations to all staff.

### Study limitations

Our definitions of exposure were policy-driven and aligned with the WHO definitions at the time the outbreak occurred. These definitions of high-risk contacts were based on a human source and did not consider the possible risk from recently contaminated surfaces, which may have affected our risk classification. There was incomplete data for medical students but not statistically significant. A minority of staff were not tested for antibody so seroconversion could not be determined in this group. For cases detected by seroconversion, we cannot entirely exclude unknown, asymptomatic contacts outside of the outbreak setting that may have been the source of infection. The study’s generalizability may be limited by unique institutional policies and regional outbreak dynamics.

## Conclusion

Management of SARS-CoV-2 outbreaks requires a multi-pronged IPC approach focusing on strict compliance with social distancing, use of PPE, hand hygiene and environmental cleaning. The full delineation of an outbreak requires the use of contact tracing, and the correlation of epidemiological information with Ct values, whole genome sequencing and serology results. Addressing healthcare worker non-compliance with behavioral and social IPC measures is a critical component in outbreak management, particularly in a non-patient facing setting s and should be a key component of any control strategies. These should be devised taking into consideration the local institutional culture Good leadership, a culture of speaking up for patient safety and measures to link individual HCW IPC practices to their annual professional practice evaluations are effective measures to prevent further outbreaks.

## Data Availability

The original contributions presented in the study are included in the article/supplementary material, further inquiries can be directed to the corresponding author.
